# Multilocus Phylogenetics Show High Intraspecific Variability within *Fusarium avenaceum*

**DOI:** 10.3390/ijms12095626

**Published:** 2011-08-31

**Authors:** Tomasz Kulik, Agnieszka Pszczółkowska, Maciej Łojko

**Affiliations:** Department of Diagnostics and Plant Pathophysiology, University of Warmia and Mazury, Plac Łódzki 5, 10-957, Olsztyn, Poland; E-Mails: agnieszka.pszczolkowska@uwm.edu.pl (A.P.); macieklojko@wp.pl (M. Ł.)

**Keywords:** *Fusarium avenaceum*, phylogeny

## Abstract

*Fusarium avenaceum* is a common soil saprophyte and plant pathogen of a variety of hosts worldwide. This pathogen is often involved in the crown rot and head blight of cereals that affects grain yield and quality. *F. avenaceum* contaminates grain with enniatins more than any species, and they are often detected at the highest prevalence among fusarial toxins in certain geographic areas. We studied intraspecific variability of *F. avenaceum* based on partial sequences of elongation factor-1 alpha, enniatin synthase, intergenic spacer of rDNA, arylamine N-acetyltransferase and RNA polymerase II data sets. The phylogenetic analyses incorporated a collection of 63 *F. avenaceum* isolates of various origin among which 41 were associated with wheat. Analyses of the multilocus sequence (MLS) data indicated a high level of genetic variation within the isolates studied with no significant linkage disequilibrium. Correspondingly, maximum parsimony analyses of both MLS and individual data sets showed lack of clear phylogenetic structure within *F. avenaceum* in relation to host (wheat) and geographic origin. Lack of host specialization indicates no host selective pressure in driving *F. avenaceum* evolution, while no geographic lineage structure indicates widespread distribution of genotypes that resulted in nullifying the effects of geographic isolation on the evolution of this species. Moreover, significant incongruence between all individual tree topologies and little clonality is consistent with frequent recombination within *F. avenaceum.*

## 1. Introduction

*Fusarium avenaceum* is a widely-distributed soil saprophyte and plant pathogen of a variety of hosts [1]. This species is more common in temperate areas although its increased prevalence has also been reported in warmer regions throughout the world [2–4]. *F. avenaceum* is often involved in crown rot and head blight of barley and wheat [5]. This species is the main contaminant of grain with enniatins [6,7]. These cyclic hexadepsipeptides have antimicrobial, insecticidal and phytotoxic activities and their high cytotoxicity on mammalian cells has been reported in *in vitro* experiments [8–10].

Yli-Mattila *et al*. [11] revealed the closest genetic relationship of *F. avenaceum* to isolates identified as *F. arthrosporioides*, *F. anguioides* and morphologically distinct *F. tricinctum*. Based on an analysis of combined *TUB* (β-tubulin), *ITS* (internal transcribed spacer) and *IGS rDNA* (intergenic spacer of rDNA) sequences, *F. avenaceum.*, *F. arthrosporioides* and *F. anguioides* isolates were resolved as five separate groups indicating conflict between phylogenetic analysis and the morphological species concept [11]. Furthermore, studies of Satyaprasad *et al*. [12] underlined limited clonality within *F. avenaceum* by showing a large number of vegetative compatibility groups (VCGs) and random amplified polymorphic DNA (RAPD) groups of European isolates. Similarly, recent phylogenetic studies by Nalim *et al*. [13] based on *EF1α* (elongation factor-1 alpha), *TUB* and *IGS rDNA* data sets showed that *F. avenaceum* isolates pathogenic to lisianthus in the US were not monophyletic or clonal. This situation is characteristic for fungi that reproduce sexually. However, no sexual stage of *F. avenaceum* has been identified, although MAT-1 and MAT-2 mating types have been identified [14] and are transcribed [15]. The studies of Satyaprasad *et al*. [12] and Nalim *et al*. [13] did not find clear geographic or host type basis of groupings within *F. avenaceum*. Moreover, pathogenicity tests showed that *F. avenaceum* isolates can cause disease lesions on more than one plant species [12,13]. To date, little is known about the genetic variability of *F. avenaceum* associated with head blight of wheat despite its high prevalence and diverse geographic range. The aim of this study was to: (i) determine the phylogenetic relationships among 63 *F. avenaceum* isolates with respect to host (wheat) and geographic origin; (ii) determine whether distinct evolutionary lineages are present within *F. avenaceum*; (iii) determine recombination events within *F. avenaceum*. MLS (multilocus sequence analysis) used in this study incorporated data sets of *EF1α*, *IGS rDNA* and *RPB2* (RNA polymerase II) that are widely used in fungal phylogenetics [13,16–20]. We also incorporated a partial sequence of *ESYN1* (enniatin synthase) involved in enniatin synthesis by *F. avenaceum* and a partial sequence of *NAT2* (arylamine N-acetyltransferase) [16] which belongs to the *NAT* gene family, encoding xenobiotic metabolizing enzymes in various prokaryotes and eukaryotes.

## 2. Results

### 2.1. Sequence Characterization

Five datasets, *EF1α*, *ESYN1*, *IGS rDNA*, *NAT2* and *RPB2* were analyzed (Table 1) in this study in order to assess intraspecific variability within a group of 63 *F. avenaceum* isolates.

Among the datasets analyzed, the percentage of variable sites varied. The *IGS rDNA* region had the highest number of variable sites (20.9%) followed by *ESYN1* (14.1%), *EF1α* (4.3%), *NAT2* (1.8%) and *RPB2* (0.2%). Similarly, the percentage of parsimony informative sites also varied between data sets analyzed. The *IGS rDNA* region had the highest number of parsimony informative sites (16.7%) followed by *ESYN1* (12.7%), *EF1α* (2.9%) and *NAT2* (1.4%). No parsimony informative sites within *RPB2* were detected. MLS had 5.4% variable sites and 4.1% parsimony informative sites. Among the *F. avenaceum* collection tested, 11 *EF1α*, 13 *ESYN1*, 29 *IGS*, 6 *NAT2* and 2 *RPB2* haplotypes were identified. MLS revealed 51 haplotypes within the group of 63 *F. avenaceum* isolates studied. Haplotype diversity (0.991) and nucleotide diversity (0.01395) for the MLS data set indicated a high level of genetic variation within the group of isolates. Overall, there was no significant (Zns = 0.0951, *P* > 0.05) linkage disequilibrium for the 95 parsimony informative polymorphic sites within the *F. avenaceum* collection.

### 2.2. Maximum parsimony (MP) Analysis of Individual and Combined Gene Sequences

**Figure 1:**
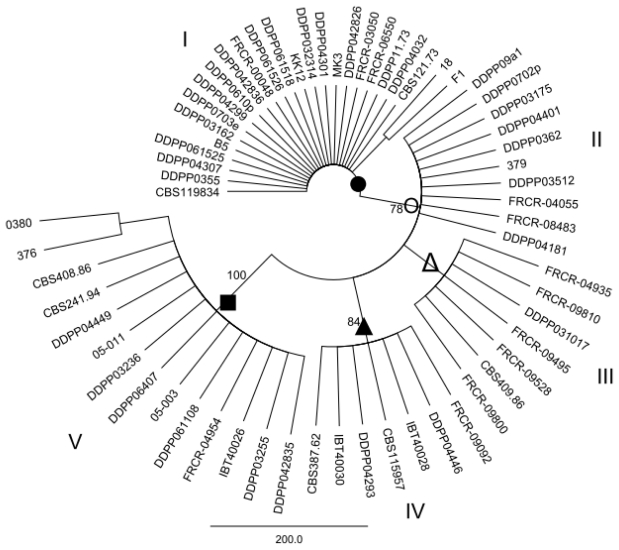
Bootstrap consensus tree inferred from the MP analysis of *EF1α* sequence data. Values at branches indicate branch support, with bootstrapping percentages based on maximum parsimony analysis. Bootstrap values ≥70% are indicated. *EF1α* groups were marked with individual symbols (•○Δ▴■) in order to visualize discord (see Figures 2, 3 and 4) between *EF1α* as an example and *ESYN1*, *IGS rDNA* and *NAT2*.

**Figure 2:**
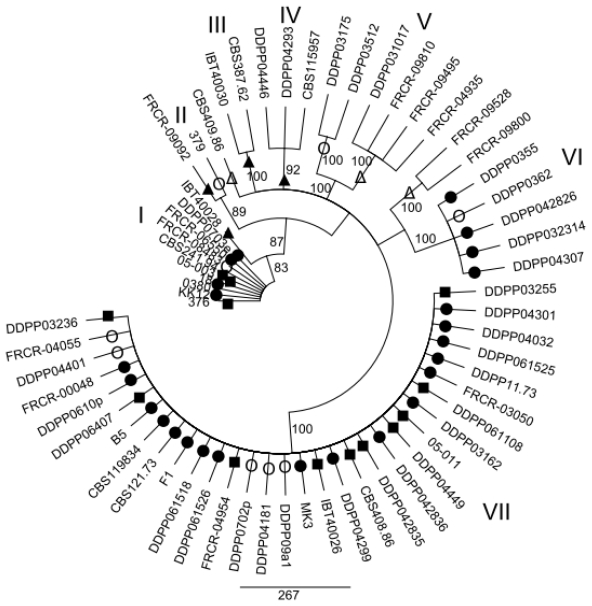
Bootstrap consensus tree inferred from the MP analysis of ***ESYN1*** sequence data. Values at branches indicate branch support, with bootstrapping percentages based on maximum parsimony analysis. Bootstrap values ≥70% are indicated. Symbols •○Δ▴■ represent five *EF1α* groups shown in Figure 1 in order to visualize discord between *EF1α* and *ESYN1* as an example.

**Figure 3:**
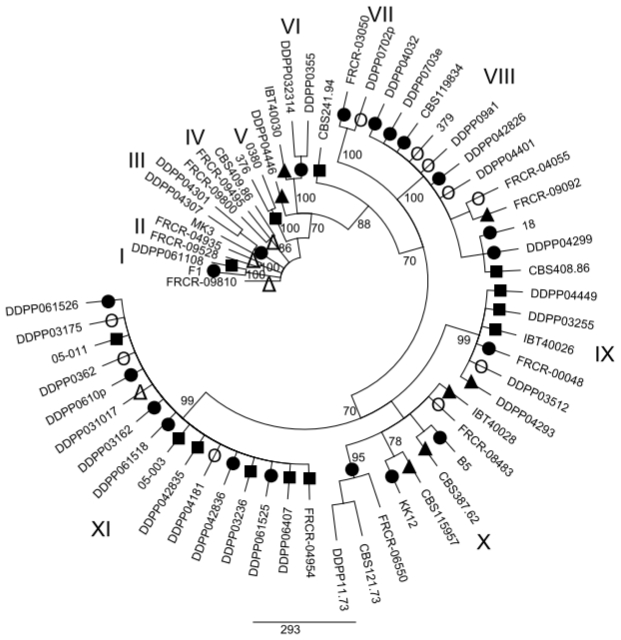
Bootstrap consensus tree inferred from the MP analysis of *IGS rDNA* sequence data. Values at branches indicate branch support, with bootstrapping percentages based on maximum parsimony analysis. Bootstrap values ≥70% are indicated. Symbols •○Δ▴■ represent five *EF1α* groups shown in Figure 1 in order to visualize discord between *EF1α* and *IGS rDNA* as an example.

**Figure 4:**
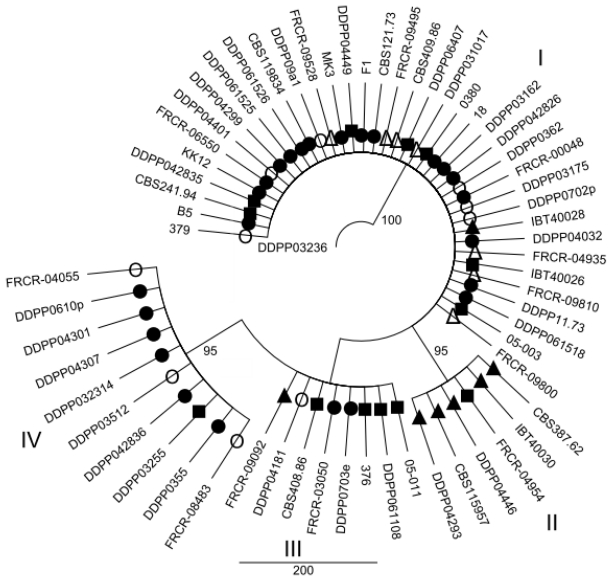
Bootstrap consensus tree inferred from the MP analysis of *NAT2* sequence data. Values at branches indicate branch support, with bootstrapping percentages based on maximum parsimony analysis. Bootstrap values ≥70% are indicated. Symbols •○Δ▴■ represent five *EF1α* groups shown in Figure 1 in order to visualize discord between *EF1α* and *NAT2* as an example.

**Figure 5:**
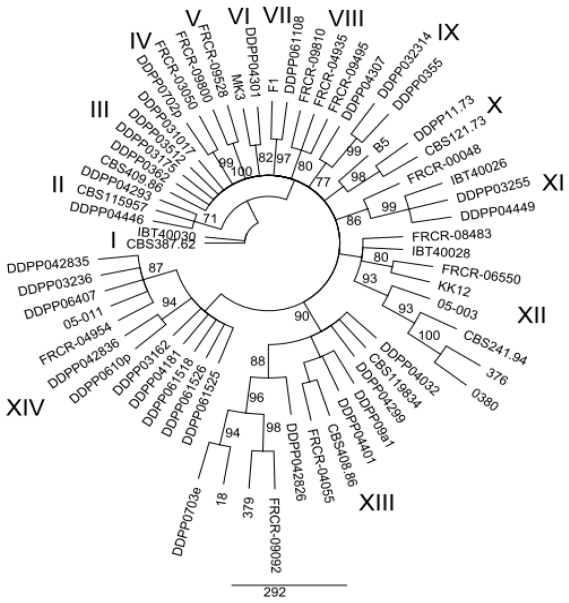
Bootstrap consensus tree inferred from the MP of combined data set (*EF1α*, *ESYN1*, *IGS rDNA*, *NAT2* and *RPB2*). Only values of the main internal branches are shown and indicate branch support, with bootstrapping percentages based on maximum parsimony analysis. Bootstrap values ≥70% are indicated.

MP analysis of the *EF1α*, *ESYN1*, *IGS rDNA*, *NAT2* data sets and MLS were performed in order to determine whether intraspecific groups exist within *F. avenaceum* (Figures 1–5). MP analysis of *RPB2* was not conducted since only 2 haplotypes were identified within this gene. Analysis of the individual *EF1α*, *ESYN1*, *IGS rDNA*, *NAT2* data sets data set revealed 5, 7, 11 and 4 main groups, respectively (Figures 1–4), however bootstrap values of some groups were not strongly supported. The groups identified within the individual data sets were not correlated with wheat as the host; however, little correlation according to origin of the isolates was observed. For example, *EF1α* groups IV and V included only isolates from Europe and one isolate (CBS 387.62) from Turkey. *IGS rDNA* groups I, III, V, VI and XI only included isolates from Europe, while three isolates from group IV originated from the US. The bootstrap consensus tree inferred from MLS analysis of the combined data set resolved 14 major groups within the collection of *F. avenaceum* isolates tested (Figure 5). Two isolates, CBS 387.62 and IBT 40030 formed outgroup of the tree. A moderate correlation between the groups and the origin of the isolates can be observed in the MLS tree. 30 isolates formed eight groups related to their origin. Groups II, VI, VII, IX, X and XIV included only European isolates, while groups V and VIII included isolates only originating from the US.

### 2.3. Partition Homogeneity Test

The partition homogeneity test showed significant incongruence in the phylogenetic signal between the five data sets (*P* = 0.002). Furthermore, after the *ESYN1* gene was excluded from the analysis the combined *EF1α*, *RPB2*, *IGS rDNA* and *NAT2* was significantly incongruent (*P* = 0.001).

## 3. Discussion

In this study, high intraspecific variability within *F. avenaceum* was observed with no significant linkage disequilibrium. MLS and analyses of the individual *EF1α*, *ESYN1*, *IGS rDNA* and *NAT2* data sets identified intraspecific groups within the collection of *F. avenaceum* isolates studied. Among the 63 isolates analyzed, 41 were associated with wheat, however, no clear link between phylogenetic groups and wheat was observed. Lack of host specialization indicates host selective pressure is not driving *F. avenaceum* evolution. Previous work by Satyaprasad *et al*. [12] did not reveal a clear separation of groups within *F. avenaceum* in relation to hosts such as lupin and wheat, based on restriction fragment length polymorphism (RFLP) and RAPD analysis. Correspondingly, subsequent studies by Nalim *et al*. [13] indicated that *F. avenaceum* isolates from lisianthus are not phylogenetically distinct from those isolated from other hosts. Recent phylogenetic studies of other *Fusarium* species such as *F. culmorum* [17], *F. poae* [18,19] and *F. pseudograminearum* [20] showed that intraspecific groups within these morphospecies are not clearly associated with a particular geographic area. Similarly, no such association was found in *F. avenaceum* [13]. Widespread distribution of local fungal genotypes is most probably the consequence of long-distance transport of plant materials that resulted in nullifying the effects of geographic isolation on the evolution of this species. In this study, moderate correlation between phylogenetic groups and the origin of the *F. avenaceum* isolates was detected based on the MLS tree, however, this may be a reflection of the isolate collection used in this work. Although isolates analyzed in this study originated from different geographic areas, the majority were of European origin. Consistent with previous reports [12,13], limited clonality within *F. avenaceum* was observed in this study. From the collection of *F. avenaceum* isolates analyzed, only 12 (19%) isolates could be classified in five separate clonal groups (Table 2).

In order to assess the genotype distribution within single wheat heads, 2–3 isolates were recovered for analysis from 6 single wheat samples (Table 2). Interestingly, among this group of 14 isolates only two isolates (DDPP 061525 and DDPP 061526) appeared to be clonal. This result showed that the distribution and co-occurrence of different *F. avenaceum* genotypes is not restricted to a single field sample. Little clonality of *F. avenaceum* could suggest sexual reproduction, although a teleomorph has not been reported from laboratory or field studies. However, MAT-1 and MAT-2 mating types have been detected [14] and they are transcribed in the genome of *F. avenaceum* [15]. Recombination events generate incongruence between individual tree topologies, whereas under a model of clonality, the topologies of all trees should be congruent [21]. To address the question of reproductive mode in *F. avenaceum*, the partition homogeneity test was performed to assess whether the gene genealogies for the five different loci were significantly different from each other. Significant incongruence (*P* = 0.002) in phylogenetic signal between all combined data sets is consistent with recombination within *F. avenaceum.* Furthermore, the high level of genetic variation detected and the lack of significant linkage disequilibrium in the MLS data suggest recombination rather than a predominantly clonally-propagated species. Incongruence in phylogenetic signal between genes can also suggest different evolutionary histories or evolutionary origins of genes. Several processes, including incomplete lineage sorting, variable evolutionary rates or hybridization could generate differences in tree topologies [20]. Some clusters involved in secondary metabolism appear to have moved into fungal genomes by horizontal gene transfer from either prokaryotes or other fungi [22]. Evidence that trichothecene metabolite profiles are not well correlated with evolutionary relationships within the *F. graminearum* clade has been previously reported [23]. Discord between *ESYN1* or *TRI5* data sets and *EF1α, RPB2* and *IGS rDNA* has also been documented within *F. poae* [18]. However, these results showed that after the *ESYN1* gene was excluded from the analysis the combined *EF1α*, *RPB2*, *IGS rDNA* and *NAT2* was still significantly incongruent (*P* = 0.001). In conclusion, results of the present study provide new insights into population biology, reproductive mode and the degree of clonality within *F. avenaceum.* The lack of significant linkage disequilibrium within *F. avenaceum* indicates that the risk of introducing genotypes representing new lineages is probably low. However, it should be noted that sexual recombination can lead to the generation of more aggressive or toxigenic strains. Thus, further phylogenetic studies are needed to monitor population changes within *F. avenaceum* to promote more informed disease control and plant breeding strategies.

## 4. Materials and Methods

### 4.1. Collection of *F. avenaceum* Isolates

All isolates analyzed in this study are listed in Table 2. CBS isolates are held in the CBS (CBS Fungal Biodiversity Centre, Utrecht, The Netherlands) fungal collection. IBT isolates were kindly provided by Dr Ulf Thrane and are held in the IBT culture collection (Center for Microbial Biotechnology (CMB), Department of Systems Biology, Technical University of Denmark). 0380, 379, 376, 05-011 and 05-003 isolates were kindly provided by Dr S. Vogelgsang (Federal Department of Economic Affairs FDEA Agroscope Reckenholz-Taenikon ART Research for Agriculture and Nature Reckenholzstrasse 191 8046 Zurich, Switzerland). 18, MK3, B5, F1 and KK12 isolates were kindly provided by Á. Szécsi (Hungarian Academy of Sciences Plant Protection Institute P.O. Box 102 H-1525 Budapest, Hungary). All FRC (Fusarium Research Center Culture Collection) isolates were kindly provided by D.M. Geiser (Department of Plant Pathology, 121 Buckhout Laboratory, The Pennsylvania State University, University Park, PA 16802, US).

26 Polish and 1 English field isolate were obtained from wheat seed samples collected during 2003–2009 and are stored in 15% glycerol at −80 °C in the fungal collection of the DDPP (Department of Diagnostics & Plant Pathophysiology, University of Warmia and Mazury in Olsztyn, Poland). The isolates were cultured on PDA (potato dextrose agar) [24] at 25 °C prior to DNA extraction. Species identity of all *F. avenaceum* isolates were confirmed by BLAST searches [25] using the *EF1α* gene sequence data to query GenBank.

### 4.2. DNA Extraction, PCR and DNA Sequencing

DNA extraction, PCR analyses and DNA sequencing were carried out as previously described [26]. Except for the *ESYN1* gene [26], all other primers used were designed in this study. Primer pairs: avef11 CGACTCTGGCAAGTCGACCA, avef12 TACCAATGACGGTGACATAG; esy1 TTCAAGGGCTGGACGTCTATG, esyave2 GTTGGTGGCCTTCATGTTCTT; igs1 GGTGGATTTGGCTGGTTTGGG, igs2 CTCCGAGACCGTTTTAGTGGG; nat211 GGAAAGCAACCTCTTTTTCTGTTA, nat22 CTCCTTCAACGCCTCCACTCTCTC; rpbave1 ACAGGCTTGTGGTCTGGTCAA, rpbave2 GGATTGACCTTTGTCTTCAATC were used for amplification of partial *EF1α*, *ESYN1*, *IGS rDNA*, *NAT2* and *RPB2* datasets*,* respectively. All sequences were deposited in NCBI database under accession numbers: *EF1α* (HQ704072-HQ704121), *ESYN1* (HQ704122-HQ704181), *IGS rDNA* (HQ704182-HQ704234), *NAT2* (HQ914964-HQ915026), *RPB2* (HQ704235-HQ704297). The sequence identity of each gene was confirmed by BLAST searches [25].

### 4.3. Phylogenetic Analyses

Sequence data were edited and then aligned using Clustal W [27] implemented in Geneious Pro 4.0.4 with the default settings [28]. Data for each gene were analyzed separately and as a combined multilocus sequence data set (MLS). Variable sites, parsimony informative sites, number of haplotypes, haplotype and nucleotide diversity were calculated using DnaSP v. 5.0 [29]. DnaSP was also used to detect linkage disequilibrium (Zns statistic) [30] within a group of isolates tested. GC % was determined using Geneious Pro 4.0.4. Maximum parsimony (MP) was conducted using PAUP* v4.0b10 [31] implemented in Geneious Pro 4.0.4 using the heuristic search option. In addition, Modeltest version 3.06 [32] with Akaike Information Criterion (AIC) [33] model selection was used to determine the nucleotide substitution model best suited to the data set. Stability of clades was assessed by 1000 MP bootstrap replications.

### 4.4. Partition Homogeneity Test

Congruence between individual gene data sets was tested using the partition homogeneity test [34] implemented in PAUP* v4.0b10 [31]. 500 replicates were analyzed in a heuristic search. The maximum number of trees was set to 1000. Invariant characters were deleted prior to analysis and 0.01 was used as the significance threshold [35].

## 5. Conclusions

Analysis of multilocus sequence (MLS) data indicated a high level of genetic variation within a group of isolates studied with no significant linkage disequilibrium. Correspondingly, maximum parsimony analyses of both MLS and individual data sets did not detect any phylogenetic structure within *F. avenaceum* in relation to host (wheat) and geographic origin. Lack of host specialization indicates host selective pressure is not driving *F. avenaceum* evolution, while no geographic lineage structure indicates widespread distribution of genotypes that resulted in nullifying the effects of geographic isolation on the evolution of this species. Significant incongruence between all individual tree topologies and little clonality is consistent with frequent recombination within *F. avenaceum.*

## Figures and Tables

**Table 1:** Sequence characteristics and phylogenetic information of individual *EF1α*, *ESYN1*, *IGS rDNA*, *NAT2*, *RPB2* and MLS datasets for *F. avenaceum* collection analyzed.

Dataset	Sequence Range	% GC	No. Variable Sites (%)	No. Parsimony Informative Sites (%)	Nucleotide Diversity	No. of Haplotypes	Haplotype Diversity
*EF1α*	510–514	53.1	22 (4.3%)	15 (2.9%)	0.00931	11	0.814
*ESYN1*	508–510	56	72 (14.1%)	65 (12.7%)	0.03303	13	0.710
*IGS rDNA*	482–516	49.1	108 (20.9%)	86 (16.7%)	0.04662	29	0.929
*NAT2*	513	50.9	9 (1.8%)	7 (1.4%)	0.00354	6	0.598
*RPB2*	518	51.3	1 (0.2%)	0	0.00006	2	0.032
MLS combined	2023–2572	51.8	138 (5.4%)	106 (4.1%)	0.01395	51	0.991

**Table 2:** List of *F. avenaceum* isolates used for phylogenetic analyses.

Isolate Number 1	Geographical Origin, Host/Habitat of Origin	*F. avenaceum* Haplotypes 2					
		*EF1α*	*ESYN1*	*IGS rDNA*	*NAT2*	*RPB2*	MLS
KK12	Hungary, wheat kerne	1	1	24	1	1	1
MK3		1	13	5	1	1	2
FRC R-03050	Australia, soil	1	13	13	5	1	3
FRC R-00048	Pennsylvania, turf	1	13	19	1	1	4
DDPP 061526 a*	Poland, wheat kernel	1	13	27	1	1	5
DDPP 0615251 a*		1	13	27	1	1	5
DDPP 03162 *		1	13	27	1	1	5
DDPP 0703e	England, wheat kernel	1	1	15	5	1	6
CBS 121.73 *	United Kingdom, *Dianthus caryophyllus*	1	13	26	1	1	7
DDPP 11.73 *	unknown	1	13	26	1	1	7
DDPP 04301 b	Poland, wheat kernel	1	13	6	6	1	8
DDPP 04307 b		1	12	6	6	1	8
DDPP 0610p	Poland, currant	1	13	28	6	1	9
DDPP 061518 a	Poland, wheat kernel	1	13	27	2	1	10
DDPP 04299 d		1	13	18	1	1	11
DDPP 042836 c		1	13	27	6	1	12
DDPP 042826 c		1	12	15	1	1	13
DDPP 04032		1	13	14	1	1	14
DDPP 0355 *		1	12	11	6	1	15
DDPP 032314 e*		1	12	11	6	1	15
CBS 119834	unknown	1	13	15	1	1	16
B5	Hungary, wheat kernel	1	13	23	1	1	17
18		3	1	18	1	1	18
F1		3	13	2	1	1	19
FRC R-06550	California, carnation	2	1	25	1	1	20
FRC R-08483	Sweden, *Salix viminalis*	5	1	22	6	1	21
FRC R-04055	South Africa, carnation	5	13	16	6	1	22
DDPP 0702p	Poland, currant	5	13	13	1	1	23
DDPP 04401	Poland, wheat kernel	5	13	15	1	1	24
379	Switzerland, wheat kernel	6	3	15	1	1	24
DDPP 04181	Poland, wheat kernel	5	13	27	5	1	25
CBS 115957	Italy, *Fagus sylvatica*	8	8	24	3	1	26
CBS 387.62	Turkey, *Camellia sinensis*	8	6	23	3	1	27
DDPP 04293 d	Poland, wheat kernel	8	7	20	4	1	28
DDPP 04446 f		8	8	9	3	1	29
IBT 40028	Denmark, wheat kernel	8	2	21	1	1	30
IBT 40030	Denmark rye kernel	8	5	10	3	1	31
FRC R-09092	Sweden, barley	9	3	17	5	1	32
DDPP 0362	Poland, wheat kernel	5	12	27	1	1	33
CBS 409.86	USA, barley kernel	7	4	7	1	1	34
FRC R-09495	California, lisianthus	7	10	7	1	1	34
FRC R-09800	Connecticut, lisianthus	7	11	7	1	1	34
DDPP 031017	Poland, wheat kernel	7	10	27	1	1	35
FRC R-09528	North Dakota, barley	7	11	4	1	1	36
FRC R-09810	Florida, lisianthus	7	10	1	1	1	37
FRC R-04935	Brazil, wheat	7	10	3	1	1	38
DDPP 03512	Poland, wheat kernel	5	9	19	6	1	39
DDPP 03175		5	9	27	1	2	40
DDPP 09a1		4	13	15	1	1	41
0380	Switzerland, wheat kernel	11	1	8	1	1	42
376		11	1	8	5	1	43
05-003		10	1	27	1	1	44
DDPP 03236 e*	Poland, wheat kernel	10	13	27	1	1	44
DDPP 042835 c*		10	13	27	1	1	44
DDPP 06407 *		10	13	27	1	1	44
05-011	Switzerland, wheat kernel	10	13	27	5	1	45
CBS 241.94	The Netherlands, *Dianthus caryophyllus*	10	1	12	1	1	46
CBS 408.86	Denmark, barley kernel	10	13	18	5	1	47
DDPP 03255	Poland, wheat kernel	10	13	19	6	1	48
IBT 40026 *	Denmark, wheat kernel	10	13	19	1	1	49
DDPP 04449 f*	Poland, wheat kernel	10	13	19	1	1	49
DDPP 061108		10	13	2	5	1	50
FRC R-04954	Germany, barley	10	13	27	3	1	51

1 Information concerning the availability of isolates analyzed in this study is given in the materials and methods section.

2 (*EF1α*) elongation factor-1 alpha, (*ESYN1*) enniatin synthase, (*IGS rDNA*) intergenic spacer of rDNA, (*NAT2*) arylamine N-acetyltransferase, (*RPB2*) RNA polymerase II, (MLS) multilocus sequence analysis. DDPP isolates recovered from the same wheat sample are designated by identical letters

a, b, c, d, e, f , respectively. Isolates belonging to the five separate clonal groups are designated by an asterisk.
